# Bidirectional mRNA transfer between *Cuscuta australis* and its hosts

**DOI:** 10.3389/fpls.2022.980033

**Published:** 2022-08-22

**Authors:** Tao Li, Yunshuang Deng, Jiaquan Huang, Jiayin Liang, Yongqin Zheng, Qian Xu, Shuting Fan, Wenting Li, Xiaoling Deng, Zheng Zheng

**Affiliations:** Guangdong Province Key Laboratory of Microbial Signals and Disease Control, South China Agricultural University, Guangzhou, China

**Keywords:** *Cuscuta australis*, sweet orange (*Citrus sinensis* “Newhall”), periwinkle [*Catharanthus roseus* (L.) G. Don], mRNAs transfer, RNA-seq

## Abstract

The holoparasitic dodder (*Cuscuta* spp.) is able to transfer mRNA and certain plant pathogens (e.g., viruses and bacteria) from the host plant. “*Candidatus* Liberibacter asiaticus,” the phloem-limited causative agent of citrus Huanglongbing, can be transferred from citrus to periwinkle (*Catharanthus roseus*) mediated by dodder. However, characterization of mRNA transport between dodder and citrus/periwinkle remains unclear. In this study, we sequenced transcriptomes of dodder and its parasitizing host, sweet orange (*Citrus sinensis* “Newhall”) and periwinkle (*Catharanthus roseus*), to identify and characterize mRNA transfer between dodder and the host plant during parasitism. The mRNA transfer between dodder and citrus/periwinkle was bidirectional and most of the transfer events occurred in the interface tissue. Compared with the citrus–dodder system, mRNA transfer in the periwinkle–dodder system was more frequent. Function classification revealed that a large number of mRNAs transferred between dodder and citrus/periwinkle were involved in secondary metabolism and stress response. Dodder transcripts encoding proteins associated with microtubule-based processes and cell wall biogenesis were transferred to host tissues. In addition, transcripts involved in translational elongation, plasmodesmata, and the auxin-activated signaling pathway were transmitted between dodder and citrus/periwinkle. In particular, transcripts involved in shoot system development and flower development were transferred between the host and dodder in both directions. The high abundance of dodder-origin transcripts, encoding MIP aquaporin protein, and *S*-adenosylmethionine synthetase 1 protein, in citrus and periwinkle tissues indicated they could play an important biological role in dodder–host interaction. In addition, the uptake of host mRNAs by dodder, especially those involved in seed germination and flower development, could be beneficial for the reproduction of dodder. The results of this study provide new insights into the RNA-based interaction between dodder and host plants.

## Introduction

Dodder (*Cuscuta australis*) is an obligate parasitic plant, which can only survive on the host plant after germination (Furuhashi et al., 2011). After dodder has parasitized the host, the haustorium directly connects with the vascular system (phloem and xylem) of the host plant (Vaughn, 2003; Yoshida et al., 2016). Interspecies plasmodesmata can also be formed between the cell wall of the searching hyphae of dodder and the host cells (Vaughn, 2003; Yoshida et al., 2016). The channels formed are mainly used for substance exchange between dodder and the host (Birschwilks et al., 2006). Dodder can absorb sugars, water, and nutrients directly from the host plant through haustoria for its survival (Clarke et al., 2019). Metabolites, proteins, mRNAs, other macromolecular substances (Haupt et al., 2001; Kim and Westwood, 2015), and certain plant pathogens, such as bacteria (Hartung et al., 2010) and viruses (Birschwilks et al., 2006; Vachev et al., 2010), can also be transferred between dodder and host. Previous study found dodder was able to transfer insect-feeding signals and defense signals between insect-attacked and non-attacked host plants (Hettenhausen et al., 2017). The large-scale translocation of mRNAs between *Cuscuta* species and its host plants has been observed and characterized (Roney et al., 2007; Kim et al., 2014; Westwood and Kim, 2017; Shahid et al., 2018; Song et al., 2021). Based on RT-PCR and microarray analysis, Roney et al. (2007) observed that the *LeGAI* transcript and nine other transcripts of tomato were present in dodder (*Cuscuta pentagona* Engelm.) grown on tomato. Four mRNAs from tomato, including the α and β subunits of *LePFP*, *RuBisCO*, and *LeGAI*, were transferred from tomato to dodder (*Cuscuta pentagona*) (David-Schwartz et al., 2008). The mRNA exchange was also observed in an *Arabidopsis*–dodder (*C. pentagona*) system, with ∼1% of the Arabidopsis mRNAs detected in dodder stems near the haustoria and ∼0.6% of the *C. pentagona* mRNAs were detected in Arabidopsis stems (Kim et al., 2014). Recently, in the green peach aphid (*Myzus persicae*)–dodder (*Cuscuta australis*)–cucumber (*Cucumis sativus*) tritrophic system, the green peach aphid feeding on dodder activated defense-related transcriptomic responses in both dodder and cucumber; in particular, a large number of mRNAs were transferred between dodder and cucumber, and between dodder and green peach aphid (Song et al., 2021). Although the mRNAs transferred between the host and dodder has certain functions in the original host, it is not clear whether they show the corresponding functions after transfer to the new host (Westwood et al., 2009). Previous studies have reported that the exogenous transferred mRNAs may play a role in regulating the growth and development of recipient plants through grafting experiments (Notaguchi et al., 2012; Zhang et al., 2016).

Dodder can transmit plant pathogens between host plants, especially vascular-limited plant pathogens, e.g., bacteria and viruses (Hosford, 1967; Hartung et al., 2010; Li et al., 2021). “*Candidatus* Liberibacter asiaticus” (CLas), a phloem-limited α-proteobacterium, is the causative agent of citrus Huanglongbing (HLB) and can be transmitted from citrus to other amenable hosts, such as periwinkle (*Catharanthus roseus*), *via* dodder. Given the rapid establishment and multiplication of CLas, and shortening latency for symptoms development induced by CLas, periwinkle is commonly used for studies of CLas (Zheng et al., 2014; Li et al., 2021; Killiny, 2022). A recent study found that the miRNA–mRNA regulatory mechanism involved in plant defense responses, regulation of secondary metabolism, and nutrient homeostasis plays an important role in periwinkle–CLas interaction (Zeng et al., 2022). In addition to acting as a transmission tool for CLas, dodder can be also used as an appropriate plant host for CLas multiplication (Li et al., 2021). The use of CLas-enriched dodder for CLas genomic and transcriptomic study generated a greater improvement in genome quality and transcriptome resolution of CLas compared with direct use of CLas-infected citrus tissue (Li et al., 2021). However, despite the advantages of the CLas–dodder–citrus/periwinkle system for research on CLas, the interaction of dodder and citrus/periwinkle remains unknown.

In this study, transcriptome sequencing of dodder and its parasitizing sweet orange (*Citrus sinensis* Osbeck “Newhall”) and periwinkle [*Catharanthus roseus* (L.) G.Don] was performed to characterize mRNA transfer between the species. The mobile mRNAs were identified and their potential functions were analyzed and discussed. The overall aim of this study was to provide novel insights into the mechanism of mRNA transfer regulation and the RNA-based interaction between dodder and citrus/periwinkle.

## Materials and methods

### Plant materials and sample preparation

**FIGURE 1:**
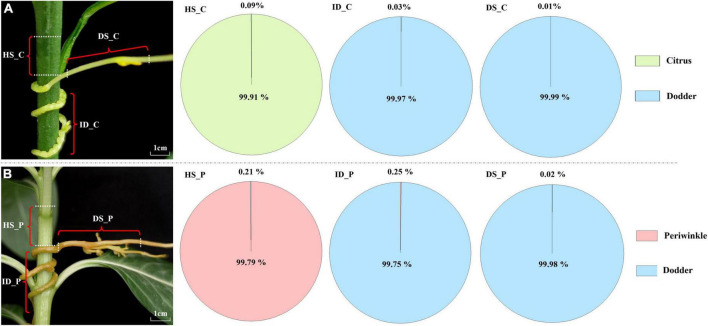
Transcriptome composition of dodder and host tissues at and near the haustoria. **(A)** Citrus-dodder system. **(B)** Periwinkle-dodder system. The pie chart depicted the proportion of the reads number for dodder and its hosts in the three distinct regions. For each host-dodder system, three types of tissues were collected. HS_C: the citrus stem above the region of attachment site; ID_C: interface region where dodder was connected to the citrus stem; DS_C: the growth extension portion of dodder stem near the region of attachment in citrus-dodder system; HS_P: the periwinkle stem above the region of attachment site; ID_P: interface region where dodder was connected to the periwinkle system; DS_P: the growth extension portion of dodder stem near the region of attachment in periwinkle-dodder system.

Three two-year-old plants of sweet orange (*Citrus sinensis* “Newhall”) with vigorous new shoots were used as the host source for dodder parasitism. Periwinkle (*Catharanthus roseus*) seeds were germinated in the greenhouse and then transferred to pots with water and fertilizer management when necessary. Dodder (*Cuscuta australis*) plants parasitized and were cultured on periwinkle after germination. Dodder plants can be used to parasitize sweet orange and periwinkle only when the tendrils extend to 7–10 cm. Three types of fresh tissue samples were collected at 20 days after dodder had successfully parasitized on two hosts: the interface region of dodder (ID, haustorium-producing portion of the dodder plant), the 1-cm-long host stem above the haustorium-producing portion of the dodder plant (HS, host stem), and the growth extension portion of the dodder stem (DS, dodder stem) (Figure 1). The tissue types from different dodder-host system were named in the following format: Tissue types _ host species (C, citrus and P, periwinkle). For example, HS_C, ID_C, and DS_C meant tissues collected from citrus-dodder system, and HS_P, ID_P, and DS_P meant tissues collected from periwinkle-dodder system. All tissue samples were wiped and disinfected with 75% alcohol, and then frozen in liquid nitrogen for RNA extraction.

### Ribonucleic acid extraction, sequencing, and analyses

Ribonucleic acid extraction was performed using the OMEGA Plant RNA Isolation Kit (OMEGA Bio-tek Co., Guangdong, China) following the manufacturer’s instructions. The quality and concentration of the RNA extract was tested with a Qubit 2.0 fluorometer (Thermo Fisher Scientific Inc., Waltham, MA, United States) and an Agilent 2100 Bioanalyzer (Agilent Technologies Inc., Santa Clara, CA, United States). RNA reverse transcription was performed using the TransScript^§^ One-step gDNA Removal and cDNA Synthesis SuperMix (TransGen Biotech, Beijing, China). For RNA sequencing (RNA-Seq), libraries were prepared by enriching eukaryotic mRNA with magnetic beads with oligo (DT) and followed by cDNA synthesis. RNA-Seq was performed on an Illumina NovaSeq 6000 platform with 150 bp paired-end reads by a commercial sequencing company. All clean sequencing data were mapped to three reference genomes (*Catharanthus roseus*, GCA_000949345.1; *Cuscuta australis*, GCA_003260385.1; *Citrus sinensis*, GCA_00031741.5) using CLC Genomic Workbench 19.0 (QIAGEN Bioinformatics, Aarhus, Denmark) with high-stringency parameters (mismatch cost = 1, insertion cost = 1, deletion cost = 1, length fraction = 1, and similarity fraction = 0.97, Non-specific matched handling = ignore). The mapped reads to non-self genomes were retrieved and used for further analyses of mobile mRNAs.

### Identification and quantitative transcriptomic analysis of mobile and non-mobile mRNAs

The number and proportion of mobile reads were calculated based on the mapping results. The transcriptomic analysis of mobile reads was performed using the RNA-Seq Analysis tool of CLC Genomic Workbench 19.0 (QIAGEN Bioinformatics, Aarhus, Denmark) with the three reference genomes and high-stringency parameters (mismatch cost = 2, insertion cost = 1, deletion cost = 1, length fraction = 1, similarity fraction = 0.97, and maximum number of hits allowed = 1). The fragments per kilobase of exon model per million mapped fragments (FPKM) were used for data normalization. The mobile and non-mobile mRNAs were identified based on transcriptomic analysis results in accordance with a previous study (Kim et al., 2014). Briefly, mRNA that detected in native tissue and not be transferred inter-species to others species through the haustoria was classified as non-mobile transcript. To identify the transcript with reliable mobility, the threshold of four mapped reads was used. mRNA with mapped reads ≥ 4 was identified as mobile mRNA. mRNAs not meeting the four reads threshold for mobility were also classified as non-mobile. The diagram of abundance and quantitative transcriptomic analysis of mobile and non-mobile mRNAs was generated by Origin 2021 (OriginLab Corp., Northampton, MA, United States).

### Homologous clustering and annotation of mobile and non-mobile mRNAs

The homologous clustering of mRNAs was performed with OrthoVenn2 (Xu et al., 2019) by selecting the plants database with the default parameters (*E*-value = 1e-2 and inflation value = 1.5). Annotation of all mRNAs was performed with Eggnog-mapper (Cantalapiedra et al., 2021). The GO (Gene Ontology) Level Count of mRNAs was assessed with TBtools software (Chen et al., 2020) and visualized with Origin 2021 (OriginLab Corp., Northampton, MA, United States). Transcript verification was performed by reads mapping and PCR analysis of four mobile mRNAs, comprising the *NsLTP* (*non-specific lipid transfer protein*) transcript of citrus, *Mito_carr* (mitochondrial carrier) transcript of periwinkle, and the *S-adomet_synt_C* (*S*-adenosylmethionine synthetase, C-Terminal Domain) transcript and *HSP 90* (Heat Shock protein) transcript of dodder. Reads mapping was performed with CLC Genomic Workbench 19.0 (QIAGEN Bioinformatics) with high-stringency parameters as described above. For PCR confirmation, primers for each mRNA were designed with Primer3Plus (Untergasser et al., 2007). All primers were listed in Supplementary Table 1.

## Results

### Transfer of mRNAs between *Cuscuta australis* and its host

The Illumina HiSeq platform generated a sequencing depth of 36∼44 million 150 paired-end reads per library with Q30 > 92 (Supplementary Table 2). The number of native and exogenous reads was calculated based on mapping with genome of three species. In the citrus–dodder system, 21,788 dodder reads (about 0.09% of the total reads) were identified in the citrus stem, whereas 8,220 citrus reads (about 0.03% of the total mapped reads) were identified in the interface tissue of the dodder attachment site (Figure 1). A total of 2,593 citrus reads (0.01% of the total reads) were identified in dodder stems (Figure 1A). Reads mapping of sequencing data from the periwinkle–dodder system revealed a higher ratio of mRNA transfer compared with the citrus–dodder system, whereas the pattern of mRNA transfer was similar to that observed in the citrus–dodder system (Figure 1B). A total of 51,959 dodder reads (0.21% of the total reads) were identified in the periwinkle stem and 58,990 periwinkle reads (0.25% of the total mapped reads) were detected in the interface tissue (Figure 1B). In addition, 5,462 periwinkle reads (0.02% of the total reads) were identified in dodder stems (Figure 1B). Reads mapping showed that mRNA transfer was bidirectional in the citrus–dodder system and the periwinkle–dodder system. The proportion of host reads in the interface tissue was higher than that observed in dodder stems, which revealed higher frequency of mRNA transfer in the haustoria region compared with that in the dodder stem.

### Identification of transcript mobility between *Cuscuta australis* and its host

**FIGURE 2:**
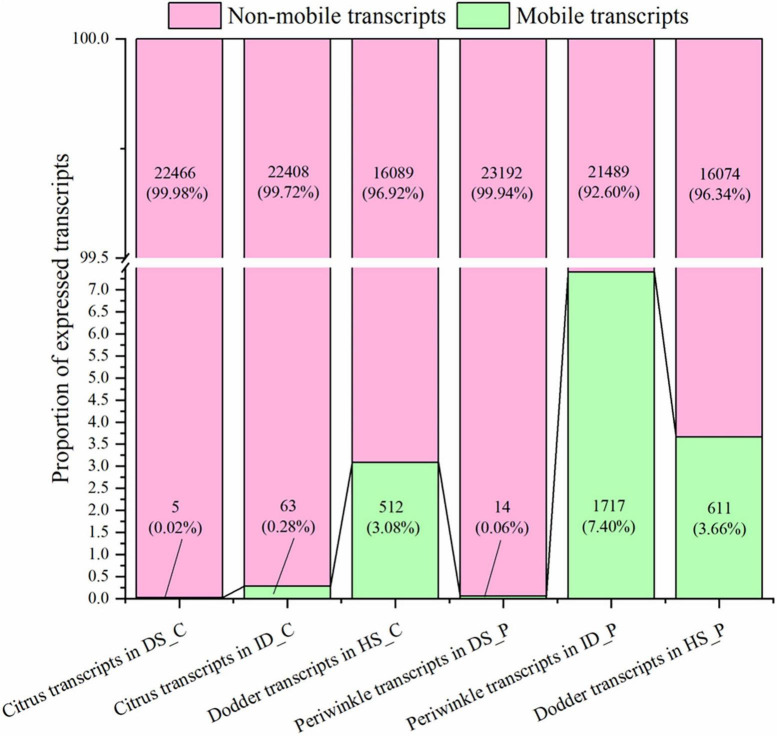
Distribution and frequency of mobile and non-mobile transcripts in different tissues. DS_C: the growth extension portion of dodder stem near the region of attachment in citrus-dodder system; ID_C: interface region where dodder was connected to the citrus stem; HS_C: the citrus stem above the region of attachment site; DS_P: the growth extension portion of dodder stem near the region of attachment in periwinkle-dodder system; ID_P: interface region where dodder was connected to the periwinkle system; HS_P: the periwinkle stem above the region of attachment site. mRNAs with mapped reads ≥ 4 were identified as mobile transcripts (represent as green bar). Transcripts that detected in native tissue and not meeting the four reads threshold for mobility were classified as non-mobile (represent as pink bar). Both the number and proportion of transcripts were listed in the corresponding bar. The diagram was generated based on mRNA abundance by Origin 2021.

Based on the high-stringency transcriptomic analysis from the three species, the dodder transcripts moving into the host stem and host transcripts moving into dodder were analyzed. To identify the transcript with reliable mobility, only transcripts with ≥ 4 mapped reads were classified as mobile. Transcripts that detected in native tissue and not meeting the four reads threshold for mobility were classified as non-mobile. In the citrus–dodder system, 63 citrus mobile transcripts (0.28% of the total expressed transcripts) were identified in the interface parasitic tissue, whereas five citrus transcripts (0.02% of the total expressed transcripts) were identified as mobile transcripts in the dodder stem tissue (DS_C) (Figure 2). In contrast, 512 transcripts (3.08% of the total expressed transcripts) were classified as dodder mobile transcripts in the citrus stem (Figure 2). Periwinkle host uptake of dodder transcripts was higher than that observed in citrus, with 611 dodder mobile transcripts (3.66% of the total expressed transcripts) identified in the periwinkle stem tissue (Figure 2). In the periwinkle–dodder system, 1,717 mobile transcripts (7.40% of the total expressed transcripts) in the interface tissue were from periwinkle and 14 transcripts (0.06% of the total expressed transcripts) were identified as periwinkle mobile transcripts in the dodder stem (Figure 2).

**FIGURE 3:**
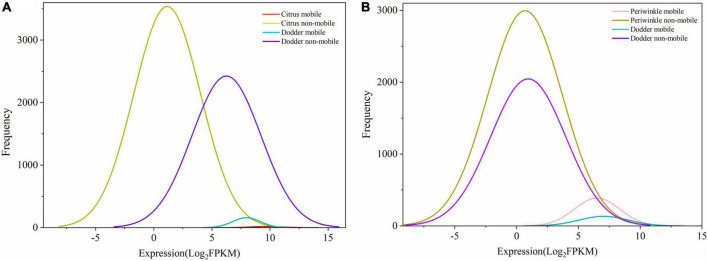
Frequency and expression levels of mobile and non-mobile transcripts in different tissues. **(A)** Citrus-dodder system. **(B)** Periwinkle-dodder system. The *x*-axis indicated the expression level of transcript represent by Log_2_FPKM. The *y*-axis indicated the number of transcripts. mRNAs with mapped reads ≥ 4 were identified as mobile mRNA. mRNAs that detected in native tissue and not meeting the four reads threshold for mobility were classified as non-mobile. The diagram was generated based on quantitative transcriptomic analysis result by Origin 2021.

Although the number of host transcripts decreased from the interface tissue to the dodder stem in the citrus–dodder system and the periwinkle–dodder system, the pattern of citrus mobile and non-mobile transcripts in the interface tissue differed from those observed in the periwinkle–dodder system (Figure 2). A significantly higher proportion of periwinkle mobile transcripts (1,717 transcripts, 7.4%) was observed in the interface tissue than the proportion of citrus mobile transcripts (63 transcripts, 0.28%) observed in the interface tissue. In addition, a higher proportion (92.60–99.98%) of non-mobile mRNAs in comparison with mobile transcripts was observed in all tissues (Figure 2). Although the number of dodder mobile transcripts was lower than dodder non-mobile transcripts in both systems, the expression abundance of most dodder mobile mRNAs was obviously higher than dodder non-mobile mRNAs (Figure 3).

### Functional clustering analysis of mobile and non-mobile mRNAs

**FIGURE 4:**
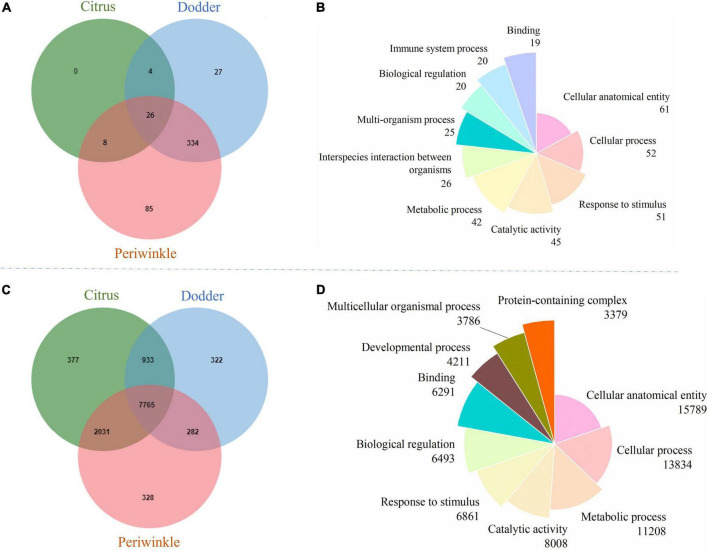
Orthologous cluster analysis and GO function annotation of mobile and non-mobile transcripts from dodder, citrus and periwinkle. **(A)** The Venn diagram showed the orthologous cluster of mobile transcripts among three species. **(B)** The pie chart showed the GOSlim terms distribution of the orthologous cluster for common mobile transcripts among three species. **(C)** The Venn diagram showed the orthologous cluster of non-mobile transcripts among three species. **(D)** The pie chart showed the GOSlim terms distribution of the orthologous cluster for common non-mobile transcripts among three species. The number of transcripts that classified to each GOSlim term was listed below the name of term. The detailed GO information of mobile and non-mobile transcripts was listed in Supplementary Table 3.

Cluster analysis of orthologs revealed 26 common mobile orthologous function clusters (Figure 4A) and 7,765 common non-mobile orthologous function clusters (Figure 4C) among citrus, periwinkle, and dodder. The enriched GO terms (> 10% of the total transcripts) of transcripts common to mobile and non-mobile transcripts among the three species were similar. Most function clusters were involved in secondary metabolism, including cellular anatomical entity, cellular process, metallic process, response to stimulus, and catalytic activity (Figures 4B,D). Detailed information on the GO analysis of mobile and non-mobile transcripts is provided in Supplementary Table 3.

**FIGURE 5:**
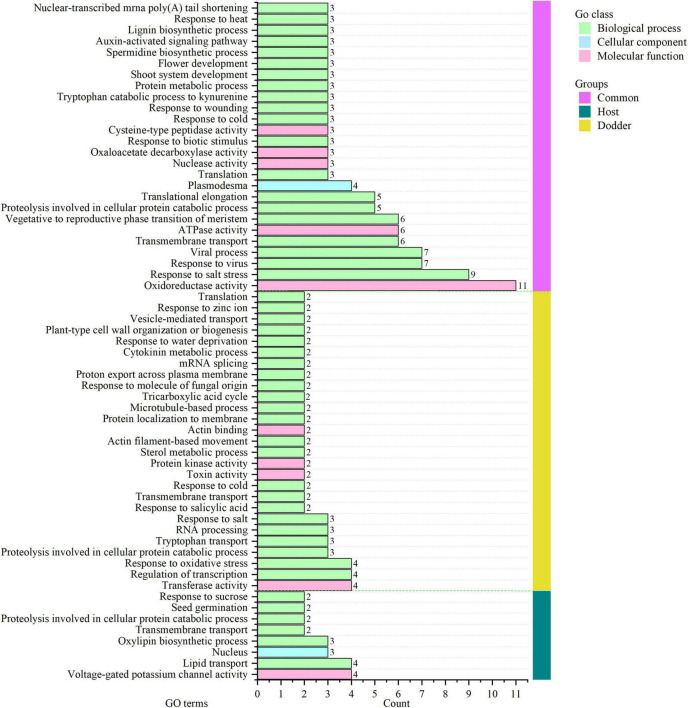
GO annotation of orthologous clusters of mobile transcripts between dodder and hosts. Common group (vertical pink bar), homologous clusters of mobile transcripts identified from dodder and citrus/periwinkle. Host group (vertical green bar), homologous clusters of mobile transcripts from citrus and periwinkle. Dodder group (vertical yellow bar), unique clusters of mobile transcripts from dodder. The count of mobile transcripts in each GO terms was indicated as the horizontal bar with the number listed in the right. The detailed GO information of mobile transcripts was listed in Supplementary Table 4.

Among 26 clusters shared by the host and dodder, transcripts involved in oxidoreductase activity, response to salt stress, response to virus, viral process, and transmembrane transport were abundant, followed by ATPase activity, and vegetative to reproductive phase transition of meristem function (Figure 5 and Supplementary Table 4). Transcripts involved in stress responses to the external environment were also transferred between dodder and citrus/periwinkle (e.g., response to biotic stimulus, response to cold, response to wounding, and response to heat) (Figure 5). In addition, transcripts associated with plant growth and development (e.g., translational elongation, plasmodesmata, and the auxin-activated signaling pathway) were transmitted between dodder and citrus/periwinkle (Figure 5). In particular, transcripts involved in shoot system development and flower development were found to be transferred from citrus/periwinkle to dodder (citrus: *LOC102622673* and *LOC102629518*, periwinkle: *Gene_57532* and *Gene_60764*) and from dodder to citrus (dodder: *DM860_000080* and *DM860_002415*) (Supplementary Table 4). Four transcripts (encoding EXORDIUM-like 2 family proteins) involved in plasmodesma function were transferred between the hosts and dodder (citrus to dodder: *LOC102625790*; dodder to citrus/periwinkle: *DM860_012892*; dodder to periwinkle: *DM860_003750*; periwinkle to dodder: *Gene_53045*) (Supplementary Table 4).

Eight mobile host-specific clusters were common to the mobile transcripts of citrus and periwinkle in the dodder stem (Figure 5). The functions of these comprised voltage-gated potassium channel activity, lipid transport, oxylipin biosynthetic process, nucleus, transmembrane transport, proteolysis involved in cellular protein catabolic process, seed germination, and response to sucrose. Among these transcripts, two host transcripts (citrus: *LOC102631491*; periwinkle: *Gene_2219*) involved in seed germination were transferred to dodder tissue (Figure 5 and Supplementary Table 4). In contrast, 27 dodder-specific mobile transcripts clusters were identified in host stems, mainly including transcripts associated with transferase activity, regulation of transcription, proteolysis involved in cellular protein catabolic process, and response to biotic and abiotic stresses (Figure 5). Notably, dodder transcripts encoding proteins associated with microtubule-based process (dodder transcripts: *DM860_013352*, *DM860_002101*) and plant-type cell wall organization or biogenesis (dodder transcripts: *DM860_011738*, *DM860_011739*) were detected in host stems (Figure 5 and Supplementary Table 4), which indicated that these transcripts may contribute to haustoria formation in the host during dodder parasitism.

**FIGURE 6:**
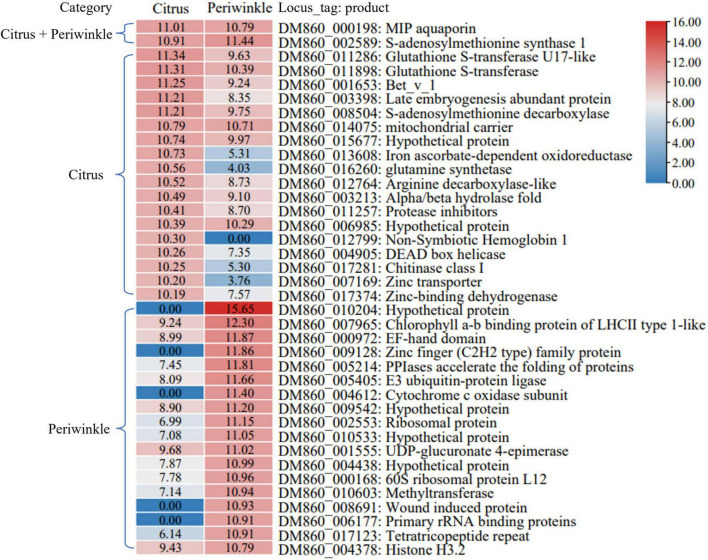
The 20 most abundant dodder mobile transcripts in host stems. The heatmap showed the normalized Log_2_FPKM values of the 20 most abundant dodder mobile transcripts detected in the citrus stem and periwinkle stem. The corresponding Log_2_FPKM of each transcript was listed in the box. Transcript that belonged to the list of the top 20 most abundant dodder mobile transcript in the citrus stem and periwinkle stem were labeled with corresponding host name in the left. The name and annotation of transcript was listed in the right.

The 20 most abundant mobile transcripts in three types of tissue were analyzed (Figure 6 and Supplementary Figure 1). The transcripts encoding a MIP aquaporin protein (*DM860_000198*) and *S*-adenosylmethionine synthetase 1 protein (*DM860_002589*) were abundant after transfer to citrus and periwinkle tissues. A higher abundance of certain dodder transcripts (e.g., *DM860_011286*, *DM860_001653*, and *DM860_003398*) in citrus tissue compared with periwinkle tissue was observed (Figure 6). A globin family transcript (*DM860_012799*) that encodes non-Symbiotic Hemoglobin 1 was highly abundant in citrus tissue but was not detected in periwinkle tissue (Figure 6). Five dodder transcripts (*DM860_010204*, *DM860_009128*, *DM860_004612*, *DM860_8691*, and *DM860_006177*) were highly abundant in the periwinkle stem but were not detected in the citrus stem (Figure 6). In addition, three host-origin transcripts encoding a non-specific lipid transfer protein (citrus: *LOC102625544*; periwinkle: *Gene_67199*, and *Gene_48474*) were highly abundant in the haustorium region of dodder (Supplementary Figure 1). Certain host transcripts (e.g., *LOC102621613* and *Gene_66120*) were highly abundant in the haustorium of dodder after their transfer, but the abundance of these transcripts was greatly reduced or undetected in the dodder stem (Supplementary Figure 1).

### Verification by *in silico* analysis and conventional polymerase chain reaction

**FIGURE 7:**
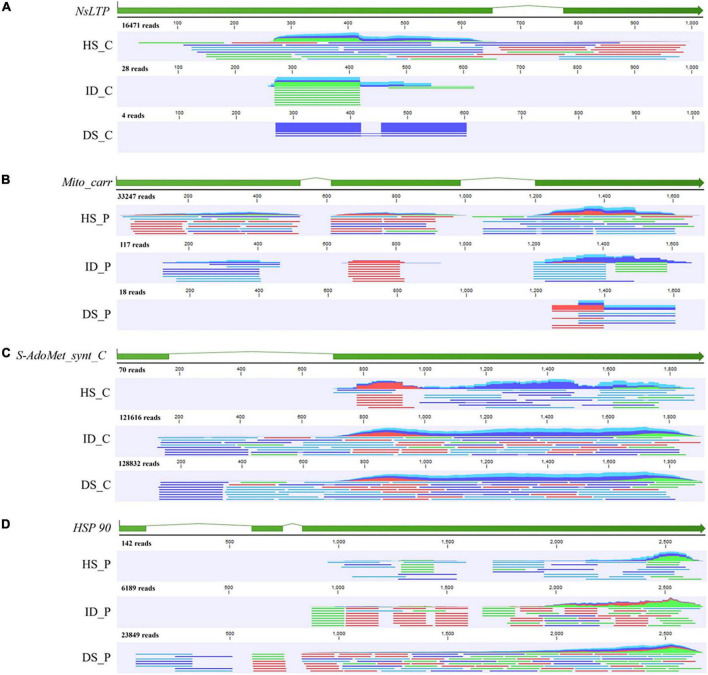
Mapping track of four selected genes with HiSeq data of different tissue types. **(A)** Mapping track of citrus *NsLTP* (*Non-specific lipid transfer protein*, citrus to dodder); **(B)** Mapping track of periwinkle *Mito_carr* (*mitochondrial carrier*, periwinkle to dodder); **(C)** Mapping track of dodder *S-AdoMet_synt_C* (*S-adenosylmethionine synthetase C-terminal domain*, dodder to citrus); **(D)** Mapping track of dodder *HSP 90* (*Heat shock protein 90*, dodder to periwinkle). Reads mapping was performed by CLC Genomic Workbench 19.0. The sample name of HiSeq data was listed in the left of mapping track. HS_C: citrus stem. ID_C: interface region of dodder’s attachment site in citrus-dodder system. DS_C: dodder stem in citrus-dodder system. HS_P: periwinkle stem. ID_C: interface region of dodder’s attachment site in periwinkle-dodder system. DS_P: dodder stem in periwinkle-dodder system. The green bar indicated the exon region of gene. In mapping track schematic diagram, the dark blue line represents the forward paired read. The light blue line represents the reverse paired read. The green line represents the forward unpaired read. The red line represents the reverse unpaired read. The total number of mapped reads was listed above the mapping track.

To further verify the RNA-Seq quality and transcript abundance after transfer, the *NsLTP* transcript of citrus, *Mito_carr* transcript of periwinkle, and *S-adomet_synt_C* transcript and *HSP 90* transcript of dodder were selected for sequence confirmation by means of transcriptome sequencing data and PCR analyses (Figure 7 and Supplementary Figure 2). Mapping results showed that the reads number decreased after transfer of the host transcript to the dodder tissue (Figure 7). A total of 16,471 reads were mapped to the *NsLTP* transcript in sequencing data for citrus stem tissue, whereas only 28 reads and four reads were mapped to the *NsLTP* transcript in sequencing data for the haustorium tissue and dodder stem, respectively (Figure 7). The number of reads mapped to the *Mito_carr* transcript decreased from 33,247 to 18 during *Mito_carr* transcript transfer to dodder from periwinkle tissue (Figure 7). A similar pattern of decrease in number of reads was observed after transfer of the dodder transcripts to host tissues. A total of 121,616 reads were mapped to the dodder *S-AdoMet_synt_C* transcript from RNA-Seq data for the interface tissue, whereas only 70 mapped reads of the *S-AdoMet_synt_C* transcript were detected in RNA-Seq data for the citrus stem. In addition, *HSP 90* reads were reduced from 6,189 to 142 after transfer from haustorium of dodder to periwinkle stem tissue.

## Discussion

The number of mRNAs transferred between dodder and the host varied among host species. In this study, the number of mRNAs transferred between dodder and periwinkle was higher than those transferred between citrus and dodder (Figure 2). A previous study similarly observed a higher number of mRNAs transferred in the *Arabidopsis*–dodder system compared with the tomato–dodder system (Westwood and Kim, 2017). This may be due to differences in the composition and content of cell walls among host plant species. During parasitism, dodder produces haustoria and secretes pectin lyase to degrade the host cell wall so that the can invade host plant tissues (Vaughn, 2003; Johnsen et al., 2015; Striberny and Krause, 2015). However, the high content of mannan and xylan in the cell wall of host plants hinders the parasitism of dodder (Johnsen et al., 2015). As a woody plant, citrus may have higher contents of mannan and xylan in the cell walls than periwinkle, a herbaceous plant. Therefore, compared with citrus, periwinkle may be more easily parasitized by dodder, which may lead to the greater abundance of transferred mRNAs.

The genetic relationship between dodder and the host may affect the number of mRNAs transferred. The relationship between dodder and the host plant can be regarded as a perfect graft system, i.e., the haustoria cells that contact host xylem strands and phloem differentiate into xylem strands and phloem cells, respectively (Leblanc et al., 2012; Yoshida et al., 2016). Although dodder can parasitize phylogenetically distant plants, based on the theory of grafting affinity, the closer the phylogenetic relationship with dodder, the more conducive to the formation of symplastic contact between dodder and the host plant after “grafting” (Pina and Errea, 2005; Milien et al., 2012). Compared with distantly related host plants, the parasitism by dodder of a phylogenetically close host plant may be more compatible by formation of fully coupled vascular bundles between dodder and the host, which provides a bridge for the transfer of mRNAs and other substances. In addition, a higher ratio of systemic long-distance movement for host mRNA in dodder stem of citrus-dodder system (0.01% reads in DS-C/0.03% reads in ID-C) compared to that in periwinkle-dodder system (0.02% reads in DS-P/0.25% reads in ID-P) indicated that the relationship of dodder and host could also affect the long-distance movement of host RNAs in dodder stem. Previous study had described an RNA signal surveillance system at the shoot apex that control the movement of phloem-mobile mRNAs and protect from viral invasion (Foster et al., 2002). A similar regulatory mechanism for mRNA movement was suggested to be exist at the interface region of host-dodder and involved in controlling the movement of host phloem-mobile mRNAs (Roney et al., 2007). The different efficiency of host mRNA movement in dodder stem between two systems could be due to the distinct mRNA regulatory mechanism in interface region between two systems.

Plasmodesmata are organelles that established cytoplasmic continuity between adjacent cells in plants (Lucas et al., 1995). As specialized channels between plant cells, besides water and various nutrients, cellular mRNA transcripts, transcription factors, short interfering RNA, microRNA, protein-encoding messenger RNAs, and viral genome were found to move from cell to adjacent cells *via* plasmodesmata channel (Ruiz-Medrano et al., 2004; Kragler, 2013; Reagan et al., 2018). In addition to the vascular system (xylem and phloem) connection between dodder and host, plasmodesmata had also found to be formed between dodder’s searching hyphae and host cells during parasitism (Vaughn, 2003; Yoshida et al., 2016), which indicated its potential function in transfer of mRNAs between host and dodder. The difference in number and integrality of plasmodesmata connection formed between citrus-dodder system and periwinkle-dodder system could affect the efficiency of mRNA transfer. In addition, the different ratio of mRNA transfers between two systems also suggested that the plasmodesmata could also play the potential roles in the selective trafficking of mRNA.

The transferred mRNA may play an important biological role in dodder–host interaction. In this study, four transcripts encoded an EXORDIUM-like 2 family protein, which is involved in plasmodesma function between hosts and dodder (citrus: *LOC102625790*; dodder: *DM860_012892*, *DM860_003750*; periwinkle: *Gene_53045*) (Figure 5 and Supplementary Table 4). The EXORDIUM-like2 protein is necessary for plants to adapt to low-carbon and low-energy conditions (Schröder et al., 2011), which is indicative of strong nutritional competition between dodder and the host in the interface region. Several host-origin transcripts (encoding cyclic nucleotide gated ion channel proteins, CNGC; periwinkle: *Gene_4761*, *Gene_16801*, and *Gene_24784*; citrus: *LOC102625781*) identified in dodder stems were involved in voltage-gate potassium channel activity (Supplementary Table 4). The CNGC proteins play an important role in plant defense response to pathogens, stress response, and growth, and development (Duszyn et al., 2019). Conversely, two dodder transcripts *DM860_000198* (encoding a MIP aquaporin) and *DM860_002589* (encoding *S*-adenosylmethionine synthetase 1) were detected at high abundance in the stems of citrus and periwinkle (Figure 6). The main role of MIP aquaporins in plants is to transport water and other small neutral molecules through cell membranes (Kapilan et al., 2018), and *S*-adenosylmethionine synthetase 1 participates in plant development and stress response (He et al., 2019). With consideration of the competitive relationship between dodder and the host, the transferred transcripts involved in substance transport, plant growth, and defense response may play a critical role in dodder–host interaction during dodder parasitism.

The high abundance of mobile transcripts that encoded proteins associated with plant defense and resistance to biotic and abiotic stresses (Figure 6) also revealed the strong interaction between species in the dodder–citrus system and dodder–periwinkle system. For example, mobile transcripts of dodder encoding Bet_v_1 protein (*DM860_001653*) and glutathione *S*-transferase (*DM860_011898*) were detected in citrus and periwinkle tissues, respectively (Figure 6). The Bet_v_1 protein is a member of the PR-10 plant disease-related protein family, which is involved in plant defense (Kim et al., 2008). Glutathione *S*-transferase is involved in intracellular transport of auxin, and can detoxify and reduce the oxidative stress response of plants by binding to glutathione (Gullner et al., 2018; Vaish et al., 2020). Furthermore, three host mobile transcripts encoding members of the non-specific lipid-transfer protein 2-like family (citrus: *LOC102625544*; periwinkle: *Gene_67199* and *Gene_48474*) were highly abundant in the interface dodder tissue in the dodder–citrus system and dodder–periwinkle system (Supplementary Figure 1). The non-specific lipid-transfer proteins are mainly involved in crucial biological processes of plant cells, such as cell membrane stability, cell wall tissue and signal transduction, and play an important role in tolerance of biotic and abiotic stresses (Liu et al., 2015). Therefore, the interaction between dodder and the host plant not only caused stimulation of the immune defense response of both dodder and the host, but also transcripts encoding components of the defense stress response were transmitted between the species as part of the parasitization. However, whether these transcripts can be translated into proteins requires further study.

The uptake of host mRNA by dodder may be utilized to promote the growth and development of dodder. Host transcripts encoding lipoxygenase (citrus: *LOC102614914* and *LOC102625429*; periwinkle: *Gene_54834*), which is involved in oxylipin biosynthesis, were identified in dodder stems (Supplementary Table 4). Lipoxygenase is a common enzyme involved in plant physiological processes, seed germination, fruit ripening, and senescence (Viswanath et al., 2020). In addition, transcripts involved in flower development (FT, FLOWERING LOCUS T, GO:0009908) were transferred between dodder and the host during parasitism (Supplementary Table 4). The FT protein is an element of the signal that induces flowering in plants (Jackson and Hong, 2012). A previous study has shown that the *FT* genes encoded by dodder may not function to activate flowering and the FT proteins synthesized by the host are able to move into dodder stems to activate dodder flowering (Shen et al., 2020). The transfer of host flower development-associated transcripts between the host and dodder may play a crucial role in the control of flowering of dodder. In addition, transcripts encoding glycosyltransferase which is associated with seed germination, were transferred from the host to dodder (citrus: *LOC102631491*; periwinkle: *Gene_2219*) (Supplementary Table 4). Glycosyltransferase participates in the regulation of plant structure and stress response, and its overexpression can enhance seed germination (Wang et al., 2020). Mobile transcripts encoding 26S proteasome regulatory subunit proteins (Citrus_mobile: LOC102622673; Dodder_mobile: DM860_000080; Periwinkle_mobile: Gene_57532) involved in shoot system development were detected in the hosts and dodder (Supplementary Table 4). In plants, the 26S proteasome regulatory subunit proteins control the size and number of cells of shoot organs, and are closely associated with shoot growth (Kurepa et al., 2009). In addition, mobile transcripts involved in the auxin-activated signaling pathway (Citrus_mobile: LOC102614737; Dodder_mobile: DM860_011898; Periwinkle_mobile: Gene_41201) were transferred between dodder and the host (Supplementary Table 4). The auxin-activated signaling pathway modulates diverse aspects of plant growth and development (Leyser, 2018). The transfer among species of transcripts required for the plant life cycle indicates the importance of their roles in dodder–host interaction.

## Conclusion

In this study, the transfer of mRNAs between dodder and its host was compared in detail by RNA-Seq when dodder parasitized a woody plant (sweet orange, *Citrus sinensis* “Newhall”) and a herbaceous plant (periwinkle, *Catharanthus roseus*). A greater magnitude of mRNA communication was detected in the periwinkle–dodder system than in the citrus–dodder system. The mRNAs transferred between citrus/periwinkle and dodder was mainly involved in secondary metabolism and stress response. The transferred dodder transcripts involved in microtubule-based processes and cell wall biogenesis may contribute to haustoria formation in the host during parasitism. Transcripts involved in shoot system development and flower development were transferred between dodder and the host. The high abundance of dodder-origin transcripts (encoding a MIP aquaporin protein and *S*-adenosylmethionine synthetase 1 protein) in host tissue may play an important biological role in dodder–host interaction. In addition, the transfer of host mRNAs to dodder tissue, especially mRNAs involved in seed germination and flower development, may be beneficial for reproduction of dodder. These results provide novel insights into the mRNA interaction between dodder and host plants.

## Data availability statement

The original contributions presented in the study are publicly available. This data can be found here: NCBI, PRJNA847378.

## Author contributions

TL, YD, XD, and ZZ conceived and designed the experiments. TL, YD, JH, JL, YZ, QX, SF, and ZZ performed the experiments. TL, YD, JH, YZ, WL, and ZZ contributed to the bioinformatic and statistical analyses. TL, YD, and ZZ prepared the figures/tables and drafted the manuscript. TL, YD, SF, and ZZ revised the manuscript. All authors have read and agreed to the published version of the manuscript.
