# Supplementary material for: Bidirectional mRNA transfer between Cuscuta australis and its hosts
Source: Front Plant Sci. 2022 Aug 22;13:980033. doi: 10.3389/fpls.2022.980033 (PMC9441868; doi:10.3389/fpls.2022.980033)
Supplement: Supplementary file 1 [file Data_Sheet_1.PDF]

Supplementary Material

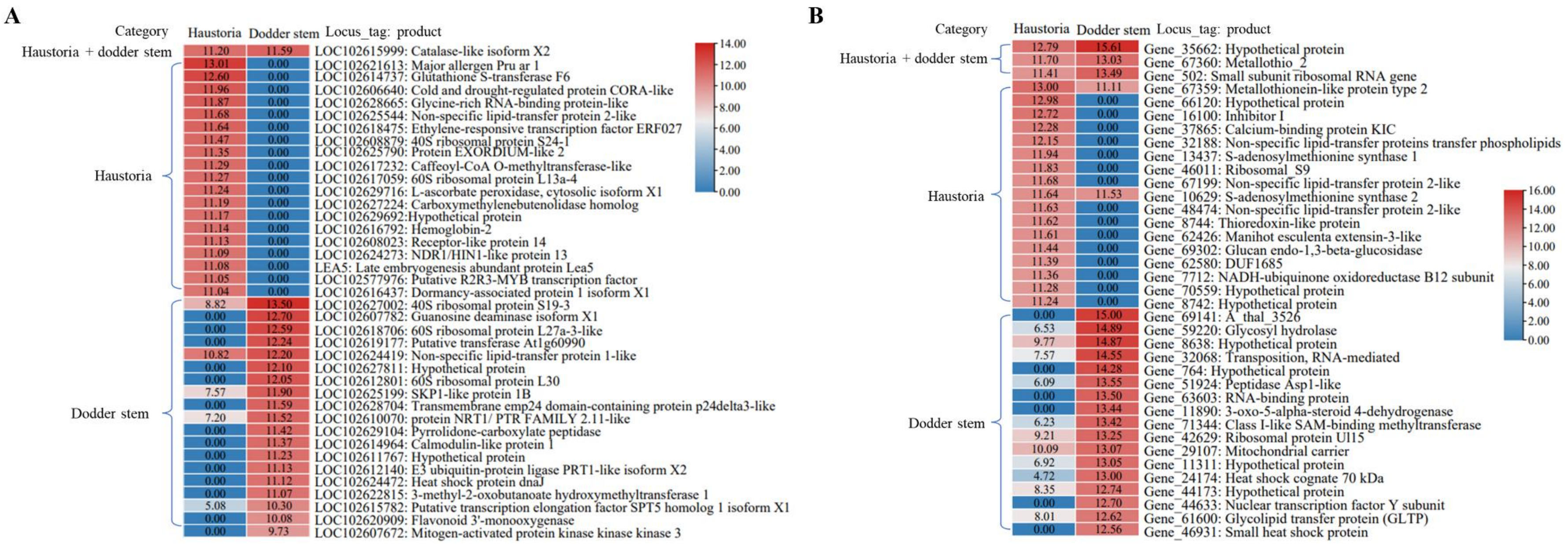

Figure S1. The top 20 high abundance of mobile transcripts of hosts. A and B indicate the top 20 transcripts with high abundance transferred from citrus to dodder and periwinkle to dodder, respectively. The heatmap showed the log2 normalized FPKM values of the top 20 high abundance transcripts in three species. The value in the box indicates the corresponding transcripts log2 FPKM value.

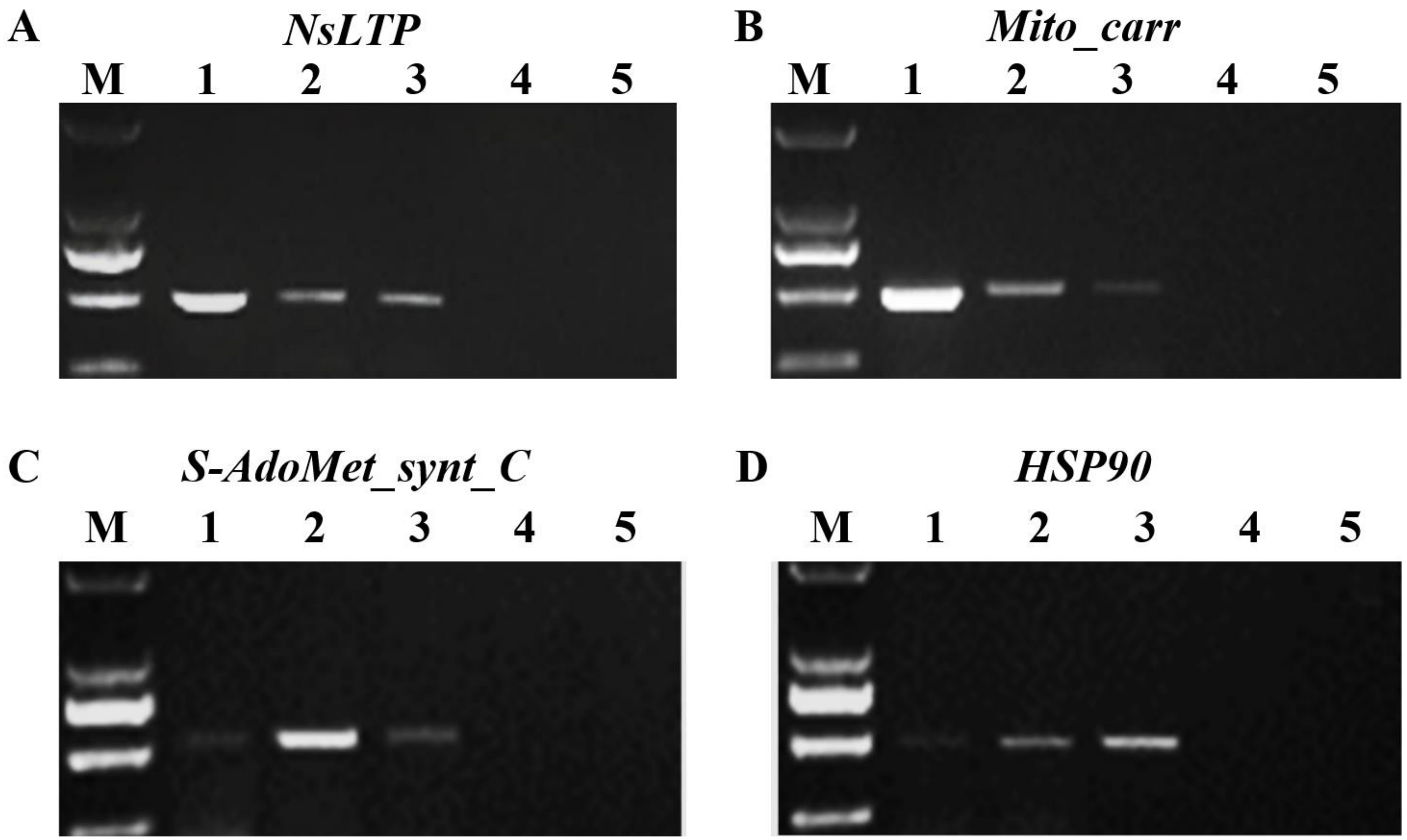

Figure S2. PCR verification result of selected transcripts in different types of tissue. A, NsLTP gene, B, Mito_carr gene, C, S-AdoMet_synt_C gene, D, HSP90 gene. Lane M shows the migration of DNA markers D2000 (Takara, China), from top to bottom: the molecular weight of which are 2000, 1000, 750, 500 and 250bp, respectively. Lane1~5 in A indicate HS_C, ID_C, DS_C, NTC (no template control) and Dodder; Lane1~5 in B indicate HS_P, ID_P, DS_P, NTC and dodder, Lane1~5 in C indicate HS_C, ID_C, DS_C, NTC and citrus, Lane1~5 in D indicate HS_P, ID_P, DS_P, NTC and periwinkle, respectively.

Table S1. Detail information of primers used for PCR verify of selected mobile transcripts

Gene name	Locus_tag	Forward primer (5‘-3’)	Reverse primer (5‘-3’)	The origin of transcript
NsLTP	LOC102624419	CTTCGACGATGCGAGGATCA	GCTCGTTTCAAGCAGTTGCA	Transcript of sweet orange transfer to dodder
Mito_carr	Gene_29107	ACCAGCAAACCACTTCCAGT	TCGCTATGGGGGTTACCAGA	Transcript of periwinkle transfer to dodder
S-AdoMet_synt_C	DM860_002589	ATGGCAAGACTCAGGTCACG	GAGGTCCAAGTTGATGGCGA	Transcript of dodder transfer to sweet orange
HSP 90	DM860_008760	CAGCCTTCTTGCTCTCACCA	GACAGCTGGAGTTCAAGGCT	Transcript of dodder transfer to periwinkle

Table S2. Summary of HiSeq data generated in the experiments.

Number	Sample name	Origin	Raw Reads	Clean Reads	Raw Base(G)	Clean Base(G)	Error Rate(%)	Q20(%)	Q30(%)	GC Content(%)
1	HS_C-1	Citrus host stem interface region tissue (citrus-dodder system)	39,878,160	38,540,824	5.98	5.78	0.03	97.47	92.78	44.06
2	HS_C-2	Citrus host stem interface region tissue (citrus-dodder system)	44,434,370	42,240,494	6.67	6.34	0.03	97.36	92.53	43.56
3	HS_C-3	Citrus host stem interface region tissue (citrus-dodder system)	46,136,224	44,247,020	6.92	6.64	0.03	97.93	93.72	43.74
4	ID_C-1	Dodder stem (citrus-dodder system)	43,076,378	41,545,892	6.46	6.23	0.03	97.55	93.14	48.97
5	ID_C-2	Dodder stem (citrus-dodder system)	45,802,366	44,109,544	6.87	6.62	0.03	97.32	92.69	48.42
6	ID_C-3	Dodder stem (citrus-dodder system)	43,500,500	41,746,446	6.53	6.26	0.03	97.41	92.90	48.29
7	DS_C-1	Dodder stem (citrus-dodder system)	39,195,532	37,997,496	5.88	5.70	0.03	97.29	92.61	48.53
8	DS_C-2	Dodder stem (citrus-dodder system)	42,674,076	41,241,742	6.40	6.19	0.03	97.48	93.00	48.28
9	DS_C-3	Dodder stem (citrus-dodder system)	42,706,942	41,473,206	6.41	6.22	0.03	97.44	92.96	47.96
10	HS_P-1	Periwinkle host stem interface region tissue (periwinkle-dodder system)	42,899,808	41,180,528	6.43	6.18	0.03	97.91	93.95	41.92
11	HS_P-2	Periwinkle host stem interface region tissue (periwinkle-dodder system)	42,325,054	41,336,918	6.35	6.20	0.03	97.89	93.94	41.67
12	HS_P-3	Periwinkle host stem interface region tissue (periwinkle-dodder system)	37,764,272	36,594,438	5.66	5.49	0.03	98.05	94.27	42.34
13	ID_P-1	Dodder stem (periwinkle-dodder system)	45,544,148	43,647,536	6.83	6.55	0.03	98.02	94.4	49.44
14	ID_P-2	Dodder stem (periwinkle-dodder system)	40,289,954	39,091,662	6.04	5.86	0.02	98.07	94.5	48.80
15	ID_P-3	Dodder stem (periwinkle-dodder system)	43,870,238	42,574,840	6.58	6.39	0.03	98	94.29	48.75
16	DS_C-1	Dodder stem (periwinkle-dodder system)	44,169,948	43,368,852	6.63	6.51	0.03	98.04	94.32	47.34
17	DS_C-2	Dodder stem (periwinkle-dodder system)	39,371,014	38,299,704	5.91	5.74	0.03	98	94.24	48.00
18	DS_C-3	Dodder stem (periwinkle-dodder system)	43,927,668	42,966,932	6.59	6.45	0.03	97.95	94.16	47.94

Table S3. GO annotation of mobile and non-mobile transcripts

Category	GO id	Description	Class	Counts
Mobile transcript	GO:0110165	Cellular anatomical entity	Cellular component	61
	GO:0009987	Cellular process	Biological process	52
	GO:0050896	Response to stimulus	Biological process	51
	GO:0003824	Catalytic activity	Molecular function	45
	GO:0008152	Metabolic process	Biological process	42
	GO:0044419	Interspecies interaction between organisms	Biological process	26
	GO:0051704	Multi-organism process	Biological process	25
	GO:0065007	Biological regulation	Biological process	20
	GO:0002376	Immune system process	Biological process	20
	GO:0005488	Binding	Molecular function	19
	GO:0140657	ATP-dependent activity	Molecular function	16
	GO:0051179	Localization	Biological process	15
	GO:0032991	Protein-containing complex	Cellular component	14
	GO:0005215	Transporter activity	Molecular function	12
	GO:0032502	Developmental process	Biological process	6
	GO:0045182	Translation regulator activity	Molecular function	5
	GO:0032501	Multicellular organismal process	Biological process	4
	GO:0023052	Signaling	Biological process	4
	GO:0098754	Detoxification	Biological process	3
	GO:0000003	Reproduction	Biological process	3
	GO:0022414	Reproductive process	Biological process	3
	GO:0016209	Antioxidant activity	Molecular function	3
	GO:0140110	Transcription regulator activity	Molecular function	3
	GO:0040007	Growth	Biological process	2
	GO:0005198	Structural molecule activity	Molecular function	2
	GO:0048511	Rhythmic process	Biological process	1
	GO:0060089	Molecular transducer activity	Molecular function	1
	GO:0110165	Cellular anatomical entity	Cellular component	15789

Category	GO id	Description	Class	Counts
Non-mobile transcripts	GO:0009987	Cellular process	Biological process	13834
	GO:0008152	Metabolic process	Biological process	11208
	GO:0003824	Catalytic activity	Molecular function	8008
	GO:0050896	Response to stimulus	Biological process	6861
	GO:0065007	Biological regulation	Biological process	6493
	GO:0005488	Binding	Molecular function	6291
	GO:0032502	Developmental process	Biological process	4211
	GO:0032501	Multicellular organismal process	Biological process	3786
	GO:0032991	Protein-containing complex	Cellular component	3379
	GO:0051179	Localization	Biological process	2973
	GO:0000003	Reproduction	Biological process	2234
	GO:0022414	Reproductive process	Biological process	2223
	GO:0023052	Signaling	Biological process	1975
	GO:0051704	Multi-organism process	Biological process	1741
	GO:0044419	Biological process involved in interspecies interaction between organisms	Biological process	1340
	GO:0140110	Transcription regulator activity	Molecular function	1303
	GO:0005215	Transporter activity	Molecular function	1211
	GO:0002376	Immune system process	Biological process	1096
	GO:0040007	Growth	Biological process	944
	GO:0140657	ATP-dependent activity	Molecular function	833
	GO:0098772	Molecular function regulator	Molecular function	406
	GO:0005198	Structural molecule activity	Molecular function	383
	GO:0060089	Molecular transducer activity	Molecular function	304
	GO:0040011	Locomotion	Biological process	174
	GO:0048511	Rhythmic process	Biological process	172
	GO:0098754	Detoxification	Biological process	155
	GO:0003774	Cytoskeletal motor activity	Molecular function	128
	GO:0016209	Antioxidant activity	Molecular function	123
	GO:0060090	Molecular adaptor activity	Molecular function	118

Category	GO id	Description	Class	Counts
Non-mobile transcripts	GO:0051703	Biological process involved in intraspecies interaction between organisms	Biological process	85
	GO:0045182	Translation regulator activity	Molecular function	71
	GO:0016032	Viral process	Biological process	62
	GO:0022610	Biological adhesion	Biological process	54
	GO:0044183	Protein folding chaperone	Molecular function	43
	GO:0140104	Molecular carrier activity	Molecular function	42
	GO:0007610	Behavior	Biological process	33
	GO:0043473	Pigmentation	Biological process	31
	GO:0019740	Nitrogen utilization	Biological process	20
	GO:0140299	Small molecule sensor activity	Molecular function	5
	GO:0110148	Biomineralization	Biological process	3
	GO:0015976	Carbon utilization	Biological process	3
	GO:0045735	Nutrient reservoir activity	Molecular function	3
	GO:0031386	Protein tag	Molecular function	3
	GO:0006791	Sulfur utilization	Biological process	2

Table S4. Orthologue clustering annotation of mobile transcripts among three species

Category	GO id	GO annotation	Protein number	Class	Locus_tag list
Host-specific clusters	GO:0005249	Voltage-gated potassium channel activity	4	Molecular function	Periwinkle_mobile: Gene_4761, Gene_16801, Gene_24784; Citrus_mobile: LOC102625781
	GO:0006869	Lipid transport	4	Biological process	Citrus_mobile: LOC102624419, LOC102625544; Periwinkle_mobile: Gene_32188, Gene_67199
	GO:0005634	Nucleus	3	Cellular component	Citrus_mobile: LOC102609575, LOC102627936; Periwinkle_mobile: Gene_59769
	GO:003140	Oxylipin biosynthetic process	3	Biological process	Citrus_mobile: LOC102614914, LOC102625429; Periwinkle_mobile: Gene_54834
	GO:0055085	Transmembrane transport	2	Biological process	Citrus_mobile: LOC102607152; Periwinkle_mobile: Gene_2705
	GO:0051603	Proteolysis involved in cellular protein catabolic process	2	Biological process	Citrus_mobile: LOC102578016; Periwinkle_mobile: Gene_44296
	GO:0009845	Seed germination	2	Biological process	Citrus_mobile: LOC102631491; Periwinkle_mobile: Gene_2219
	GO:0009744	Response to sucrose	2	Biological process	Citrus_mobile: LOC102616437; periwinkle_mobile: Gene_29919
Dodder-specific clusters	GO:0016747	Transferase activity	4	Molecular function	DM860_008850, DM860_003858, DM860_009397, DM860_014033
	GO:0006355	Regulation of transcription	4	Biological process	DM860_005322, DM860_017677, DM860_013266, DM860_005568
	GO:0006979	Response to oxidative stress	4	Biological process	DM860_007663, DM860_005458, DM860_001924, DM860_002994
	GO:0051603	Proteolysis involved in cellular protein catabolic process	3	Biological process	DM860_002972, DM860_015212, DM860_013193
	GO:0015827	Tryptophan transport	3	Biological process	DM860_013171, DM860_009561, DM860_005198
	GO:0006396	RNA processing	3	Biological process	DM860_002903, DM860_018227, DM860_001604
	GO:1902074	Response to salt	3	Biological process	DM860_014378, DM860_004706, DM860_007643
	GO:0009751	Response to salicylic acid	2	Biological process	DM860_016514, DM860_016138
	GO:0055085	Transmembrane transport	2	Biological process	DM860_007115, DM860_002324
	GO:0009409	Response to cold	2	Biological process	DM860_015313, DM860_001891
	GO:0090729	Toxin activity	2	Molecular function	DM860_004437, DM860_004438
	GO:0004672	Protein kinase activity	2	Molecular function	DM860_011652, DM860_005371
	GO:0016125	Sterol metabolic process	2	Biological process	DM860_010792, DM860_017236
	GO:0030048	Actin filament-based movement	2	Biological process	DM860_012574, DM860_010345

Category	GO id	GO annotation	Protein number	Class	Locus_tag list
Dodder-specific clusters	GO:0003779	Actin binding	2	Molecular function	DM860_004961, DM860_012058
	GO:0072657	Protein localization to membrane	2	Biological process	DM860_000436, DM860_000416
	GO:0007017	Microtubule-based process	2	Biological process	DM860_013352, DM860_002101
	GO:0006099	Tricarboxylic acid cycle	2	Biological process	DM860_002271, DM860_008844
	GO:0002238	Response to molecule of fungal origin	2	Biological process	DM860_001926, DM860_008688
	GO:0120029	Proton export across plasma membrane	2	Biological process	DM860_015973, DM860_011535
	GO:0000398	mRNA splicing	2	Biological process	DM860_010489, DM860_007915
	GO:0009690	Cytokinin metabolic process	2	Biological process	DM860_004318, DM860_007622
	GO:0009414	Response to water deprivation	2	Biological process	DM860_004136, DM860_015079
	GO:0071669	Plant-type cell wall organization or biogenesis	2	Biological process	DM860_011738, DM860_011739
	GO:0016192	Vesicle-mediated transport	2	Biological process	DM860_002588, DM860_007438
	GO:0010043	Response to zinc ion	2	Biological process	DM860_010730, DM860_010117
	GO:0006412	Translation	2	Biological process	DM860_003618, DM860_001062
Mobile mRNAs cluster between host and dodder	GO:0016705	Oxidoreductase activity	11	Molecular function	Dodder_mobile: DM860_016328, DM860_016741, DM860_000934, DM860_007891; Citrus_mobile: LOC102613827, LOC102610093, LOC102630824; Periwinkle_mobile: Gene_35189, Gene_20311, Gene_8027, Gene_9718
	GO:0009651	Response to salt stress	9	Biological process	Citrus_mobile: HAP3, LOC102618521, LOC102629716; Dodder_mobile: DM860_001159, DM860_005828, DM860_006939; Periwinkle_mobile: Gene_17102, Gene_22656, Gene_67480
	GO:0009615	Response to virus	7	Biological process	Dodder_mobile: DM860_015792, DM860_000112; Citrus_mobile: LOC102617514, LOC102626809; Periwinkle_mobile: Gene_4354, Gene_24174, Gene_17163
	GO:0016032	Viral process	7	Biological process	Citrus_mobile: LOC102616823, LOC102607198; Dodder_mobile: DM860_007483, DM860_015184; Periwinkle_mobile: Gene_14064, Gene_4081, Gene_2415
	GO:0055085	Transmembrane transport	6	Biological process	Dodder_mobile: DM860_011892, DM860_003435; Citrus_mobile: LOC102618233, LOC102624176; Periwinkle_mobile: Gene_61656, Gene_33500

Category	GO id	GO annotation	Protein number	Class	Locus_tag list
Mobile mRNAs cluster between host and dodder	GO:0016887	ATPase activity	6	Molecular function	Dodder_mobile: DM860_005785, DM860_005784, DM860_005786; Citrus_mobile: LOC102614353, LOC102625946; Periwinkle_mobile: Gene_52782
	GO:0010228	Vegetative to reproductive phase transition of meristem	6	Biological process	Citrus_mobile: LOC102628665, LOC102607686; Dodder_mobile: DM860_007537, DM860_015238; Periwinkle_mobile: Gene_36856, Gene_60468
	GO:0051603	Proteolysis involved in cellular protein catabolic process	5	Biological process	Dodder_mobile: DM860_006627, DM860_008018, DM860_012195; Citrus_mobile: LOC102623323; Periwinkle_mobile: Gene_70472
	GO:0006414	Translational elongation	5	Biological process	Dodder_mobile: DM860_002742, DM860_006256, DM860_006261; Citrus_mobile: LOC102613486; Periwinkle_mobile: Gene_56181
	GO:0009506	Plasmodesma	4	Cellular component	Dodder_mobile: DM860_003750, DM860_012892; Citrus_mobile:LOC102625790; Periwinkle_mobile: Gene_53045
	GO:0006412	Translation	3	Biological process	Citrus_mobile: LOC102616397; Dodder_mobile: DM860_012028; Periwinkle_mobile: Gene_2656
	GO:0004518	Nuclease activity	3	Molecular function	Citrus_mobile: LOC102622258; Dodder_mobile: DM860_009470; Periwinkle_mobile: Gene_71104
	GO:0008948	Oxaloacetate decarboxylase activity	3	Molecular function	Citrus_mobile: LOC102622357; Dodder_mobile: DM860_004100; Periwinkle_mobile: Gene_11095
	GO:0009607	Response to biotic stimulus	3	Biological process	Citrus_mobile: LOC102621613; Dodder_mobile: DM860_001653; Periwinkle_mobile: Gene_62779
	GO:0008234	Cysteine-type peptidase activity	3	Molecular function	Citrus_mobile: VPE; Dodder_mobile: DM860_007656; Periwinkle_mobile: Gene_58986
	GO:0009409	Response to cold	3	Biological process	Citrus_mobile: LOC102627293; Dodder_mobile: DM860_010798; Periwinkle_mobile: Gene_20290
	GO:0009611	Response to wounding	3	Biological process	Citrus_mobile: LOC102621646; Dodder_mobile: DM860_006645; Periwinkle_mobile: Gene_69918
	GO:0019441	Tryptophan catabolic process to kynurenine	3	Biological process	Citrus_mobile: LOC102615060; Dodder_mobile: DM860_010787; Periwinkle_mobile: Gene_23568
	GO:0019538	Protein metabolic process	3	Biological process	Citrus_mobile: LOC102631183; Dodder_mobile: DM860_010259; Periwinkle_mobile: Gene_9532
	GO:0048367	Shoot system development	3	Biological process	Citrus_mobile: LOC102622673; Dodder_mobile: DM860_000080; Periwinkle_mobile: Gene_57532
	GO:0009908	Flower development	3	Biological process	Citrus_mobile: LOC102629518; Dodder_mobile: DM860_002415; Periwinkle_mobile: Gene_60764
	GO:0008295	Spermidine biosynthetic process	3	Biological process	Citrus_mobile: LOC102608169; Dodder_mobile: DM860_012764; Periwinkle_mobile: Gene_8047

Category	GO id	GO annotation	Protein number	Class	Locus_tag list
	GO:0009734	Auxin-activated signaling pathway	3	Biological process	Citrus_mobile: LOC102614737; Dodder_mobile: DM860_011898; Periwinkle_mobile: Gene_41201
	GO:0009809	Lignin biosynthetic process	3	Biological process	Citrus_mobile: LOC102617232; Dodder_mobile: DM860_015186; Periwinkle_mobile: Gene_32282
	GO:0009408	Response to heat	3	Biological process	Citrus_mobile: LOC102608938; Dodder_mobile: DM860_010703; Periwinkle_mobile: Gene_30517
	GO:0000289	Nuclear-transcribed mrna poly(A) tail shortening	3	Biological process	Citrus_mobile: LOC102630158; Dodder_mobile: DM860_006135; Periwinkle_mobile: Gene_11430
